# Social genomics, cognition, and well-being during the COVID-19 pandemic

**DOI:** 10.1101/2023.05.31.23290618

**Published:** 2023-06-07

**Authors:** James R. Bateman, Sudarshan Krishnamurthy, Ellen E. Quillen, Christian E. Waugh, Kiarri N. Kershaw, Samuel N. Lockhart, Timothy M. Hughes, Teresa E. Seeman, Steve W. Cole, Suzanne Craft

**Affiliations:** 1. Department of Neurology, Wake Forest University School of Medicine, Winston-Salem, NC, USA;; 2. Department of Psychiatry and Behavioral Medicine, Wake Forest University School of Medicine, Winston-Salem, NC, USA;; 3. Department of Internal Medicine, Section of Gerontology and Geriatric Medicine, Wake Forest University School of Medicine, Winston-Salem, NC, USA; 4. Mental Illness Research, Education, and Clinical Center, Salisbury VA Medical Center, Salisbury, NC, USA;; 5. Department of Internal Medicine, Section of Molecular Medicine, Wake Forest University School of Medicine, Winston-Salem, NC, USA;; 6. Department of Psychology, Wake Forest University, Winston-Salem, NC, USA;; 7. Department of Preventive Medicine (Epidemiology), Northwestern University Feinberg School of Medicine, Chicago, IL, USA;; 8. Department of Epidemiology, UCLA Fielding School of Public Health, Los Angeles, CA, USA;; 9. Department of Medicine and Psychiatry and Biobehavioral Sciences, UCLA School of Medicine, Los Angeles, CA, USA

**Keywords:** social genomics, mild cognitive impairment, COVID, well-being, stress, loneliness

## Abstract

**INTRODUCTION:**

Adverse psychosocial exposure is associated with increased proinflammatory gene expression and reduced type-1 interferon gene expression, a profile known as the conserved transcriptional response to adversity (CTRA). Little is known about CTRA activity in the context of cognitive impairment, although chronic inflammatory activation has been posited as one mechanism contributing to late-life cognitive decline.

**METHODS:**

We studied 171 community-dwelling older adults from the Wake Forest Alzheimer’s Disease Research Center who answered questions via a telephone questionnaire battery about their perceived stress, loneliness, well-being, and impact of COVID-19 on their life, and who provided a self-collected dried blood spot sample. Of those, 148 had adequate samples for mRNA analysis, and 143 were included in the final analysis, which including participants adjudicated as having normal cognition (NC, *n* = 91) or mild cognitive impairment (MCI, *n* = 52) were included in the analysis. Mixed effect linear models were used to quantify associations between psychosocial variables and CTRA gene expression.

**RESULTS:**

In both NC and MCI groups, eudaimonic well-being (typically associated with a sense of purpose) was inversely associated with CTRA gene expression whereas hedonic well-being (typically associated with pleasure seeking) was positively associated. In participants with NC, coping through social support was associated with lower CTRA gene expression, whereas coping by distraction and reframing was associated with higher CTRA gene expression. CTRA gene expression was not related to coping strategies for participants with MCI, or to either loneliness or perceived stress in either group.

**DISCUSSION:**

Eudaimonic and hedonic well-being remain important correlates of molecular markers of stress, even in people with MCI. However, prodromal cognitive decline appears to moderate the significance of coping strategies as a correlate of CTRA gene expression. These results suggest that MCI can selectively alter biobehavioral interactions in ways that could potentially affect the rate of future cognitive decline and may serve as targets for future intervention efforts.

## Introduction

Alzheimer’s disease (AD) is a progressive neurodegenerative condition that is a common cause of both mild cognitive impairment (MCI) and dementia.^1,2^ Psychosocial risk and resiliency factors can modulate the rate of subsequent cognitive decline in the context of developing neuropathologic changes in the brain, and many of these factors may be modifiable.^3^ Key to developing interventions to harness such resilience effects is identifying the specific psychosocial processes that impact the biology of cognitive decline.

Research in those with normal cognition (NC) has identified some psychobiological pathways through which psychosocial factors may impact biological processes relevant to cognitive function. One such molecular pathway is the conserved transcriptional response to adversity (CTRA).^4^ The CTRA is a pattern of leukocyte gene expression that has been observed across species (i.e., conserved) in response to a host of adverse social conditions. The CTRA transcriptional pattern involves an increase in expression of proinflammatory genes (e.g., *IL1B, IL6, TNF*) and a decrease in the expression of type I interferon response genes (e.g., *IFI-, OAS-* and *MX*-family genes) in response to fight-or-flight stress signaling from the sympathetic nervous system (SNS). A proposed evolutionary explanation for such a response is the necessity to pivot to an anti-microbial, and away from the anti-viral, state of the immune system during periods of acute threat.^5^ Under ancestral conditions, activation of the SNS would have predicted an increased risk of wound-associated bacterial infections and thus support wound healing. In modern conditions, chronic low-level threat produces chronic activation of pro-inflammatory genes, which can contribute to the pathogenesis of a host of common chronic conditions such as neurodegenerative disorders such as Alzheimer’s disease and related dementias,^6^ cardiovascular disease, and neoplastic disorders^7,8^.This chronic, lowgrade inflammation also increases with age (“inflammaging”) and the CTRA is one mechanism through which conditions of chronic psychosocial adversity can alter underlying biology and potentially accelerate inflammaging-related disease processes.^9^ However, to date, few studies have evaluated CTRA risk or resilience processes in the context of cognitive aging.

The CTRA was first identified in the context of loneliness,^10^ and subsequent work linked these effects to reduced levels of eudaimonic well-being, or a sense of purpose and meaning in life.^11^ Eudaimonic well-being is distinguishable from hedonic well-being, which is the summation of positive affective experiences in a person’s life.^12^ In the context of cognitive aging, Boyle and colleagues in the Rush Memory and Aging Project (MAP) have previously linked a sense of purpose in life to variations in cognitive aging:^13^ participants with a greater sense of purpose in life were found to have a lower risk of both AD and MCI over a 7-year follow-up period. The biological mechanism through which a sense of purpose relates to cognitive decline is not yet known. However, given the potential role of inflammatory biology in cognitive aging, the CTRA-associated inflammatory biology may represent one mechanism through which psychosocial factors (i.e., a sense of purpose) could affect either risk or resiliency to cognitive decline.

In the present study, we conducted genome-wide transcriptional profiling of dried blood spots collected during a period of significant psychosocial stress - the first 2 years of the COVID-19 pandemic – and examined the links between CTRA gene expression and several psychosocial risk and resilience factors, including loneliness, perceived stress, and both hedonic and eudaimonic well-being. Analyses focused on understanding how the psychosocial correlates of CTRA gene expression may be similar to or different between those with normal cognition and those with mild cognitive impairment.

## Methods

### Participants

All participants were previously enrolled in the Alzheimer’s Disease Clinical Core (Clinical Core) cohort of the Wake Forest Alzheimer’s Disease Research Center (WF ADRC) and underwent standardized evaluations in accordance with the National Alzheimer’s Coordinating Center (NACC) protocol for data collection which meets Uniformed Data Set (UDS) requirements. Specific inclusion and exclusion criteria for the Clinical Core are described elsewhere.^14^ Cognitive adjudication at yearly Clinical Core study visits using clinical and cognitive assessment and brain MRI provides a cognitive diagnosis of NC, MCI, or dementia. For the purposes of this study, only participants with NC or MCI were eligible for study inclusion. The determination of mild cognitive impairment was made using clinical criteria according to NACC guidelines as described by Petersen and Morris.^15^ To address early pandemic concerns of in-person study visits, both questionnaire responses and dried blood spot collection were designed to be collected remotely. All study procedures were approved by the Wake Forest Baptist Health Institutional Review Board. Written informed consent was obtained for all participants and/or their legally authorized representative. Questionnaires were administered via telephone between February 15 and July 21, 2021. Dried blood spot collection occurred a median of 8 days (range: −40 to 136 days) following the questionnaire completion.

### Perceived Stress

The 10-item Perceived Stress Scale (PSS) is a questionnaire that is an index of an individual’s perception of stress over the past month.^16^ Stress was operationalized as finding one’s life unpredictable and uncontrollable, and feeling overloaded. The questions were answered on a Likert scale that ranged from “never” (0) to “very often” (4) after reversing the four positively stated questions. Individual items are summed to produce a total score and showed good internal reliability (α = 0.85). Higher scores reflect higher levels of perceived stress.

### Loneliness

Loneliness was assessed using the UCLA Loneliness Scale, Version 3.^17^ Participants rate statements to describe how often they feel the way described, ranging from “never” (0) to “often” (4). There were twenty statements, and nine were reverse coded according to standard instructions. A total score was computed with higher scores indicating greater feelings of loneliness and showed good internal reliability (α = 0.85).

### Coping

The Brief Coping Orientation to Problems Experienced (brief-COPE) was developed by Carver^18^ as an abbreviated version of the longer COPE, which contains 28 items that measure 14 factors of coping along a Likert scale ranging from “I have not been doing this at all” (0) to “I have been doing this a lot” (3). We added 6 questions related to positive distraction.^19^ Consistent with prior research,^20^ we performed a parallel factor analysis which identified a 3 factor solution of Support (comprised of emotional support, instrumental support, and active coping items), Distraction and Reframing (comprised of positive distraction, positive reframing, and self-distraction), and Blame and Disengagement (comprised of self-blame and behavioral disengagement). Non-participating factors were denial, substance use, venting, planning, humor, religion, and acceptance.

### Hedonic and Eudaimonic Well-Being

The Mental Health Continuum – Short Form (MHC-SF)^12^ is a 14-item questionnaire derived from a 40-item questionnaire.^21^ The MHC-SF was designed to measure hedonic and psychological well-being as conceptualized by Ryff and social well-being as conceptualized by Keyes.^22,23^ Respondents were asked to answer questions about the degree to which they’ve felt a given way over the past month ranging from “never” (0) to “every day (5). Three questions were summed for hedonic well-being (HWB), five for social well-being (SWB), and six for psychological well-being (PWB). SWB and PWB together make up eudaimonic well-being (EWB). The overall internal reliability of the MHC-SF was good (α = 0.89).

### COVID-19 Specific Experiences

Two questionnaires were given to assess the impact of COVID-19 on participants. We administered by telephone the participant version of the COVID-19 Impact Survey, version 1^24^ from the NACC that was initially released in the Summer of 2020. This questionnaire captured information regarding COVID-19 exposure, medical consequences, impact on psychosocial factors, and perceived cognitive, psychiatric, and behavioral consequences. We supplemented this questionnaire with questions from the Questionnaire for Assessing the Impact of COVID-19 Pandemic and Accompanying Mitigation Efforts on Older Adults (QAICPOA).^25^ The QAICPOA was developed specifically to assess the impact of COVID-19 on older adults and includes questions regarding diagnosis, symptoms, actions taken because of the pandemic, changes in contact and communication. Some questions included in these forms were discrete dates (e.g., dates of COVID diagnosis), others were yes/no responses to questions regarding types of care accessed. For the purposes of this study, three questions were included in the analysis, all from the COVID-19 Impact Survey. Questions 7 (worry about COVID-19 infection/reinfection), 8 (isolated or cut off from family and friends due to COVID-19), and 9 (disruption to everyday life due to COVID-19). Each of these questions were answered on a five point Likert scale ranging from “not at all” (one) to extremely (five).

### Dried Blood Spot Collection

After questionnaires were collected, participants were mailed a remote collection kit for selfcollection of dried blood spots. Training materials were adapted for use in our cohort from Allen and colleagues.^26^ Participants were sent all necessary materials and a printed instruction booklet with instructions on specimen collection. Blood spots were placed directly on a standardized filter paper commonly used for neonatal screening (Whatman #903, GE Healthcare, Piscataway, NJ). Pictorial examples of both good and bad dried blood spot collection were provided. Participants were instructed to allow the collection card to sit for four hours to dry, then place the folded collection card, along with a humidity detector and silica gel packs, in a provided gas permeable bag and return to the WF ADRC in a provided return envelope.

### Measurement of Gene Expression

Dried blood spot were stored at −80°C at the WF ADRC and then shipped as a single batch on dry ice to the UCLA Social Genomics Core Laboratory for transcriptome-wide RNA profiling and CTRA gene expression analyses as previously described.^27,28^ Briefly, RNA was extracted (Qiagen RNeasy), converted into cDNA using a high-efficiency mRNA-targeted reverse transcription system (Lexogen QuantSeq 3’ FWD), and sequenced on an Illumina NovaSeq instrument in the UCLA Neuroscience Genomics Core Laboratory, all following the manufacturers’ standard protocols for this workflow. Sequencing targeted >10 million single stranded 100-nt reads per sample (achieved median = 17.3 million), each of which was mapped to the GRCh38 reference human transcriptome using the STAR aligner (median 83% mapping rate), and quantified as gene transcripts per million total mapped reads with expression values floored at 1 transcript-per-million to suppress spurious low-range variability, log2-transformed to stabilize level-dependent variance within gene, and z-score transformed to stabilize variance across genes. Among 171 assayed samples, routine postassay data quality screening identified 7 samples with insufficient RNA sequencing reads (< 5 million), 8 additional samples with poor read mapping rates (< 70%), and 6 additional samples with poor signal-to-noise ratios (average profile correlation with other samples: r < .50), leaving a total of 150 valid RNA profiles available for analyses of CTRA. This 88% valid data yield is consistent with previous research involving genome-wide transcriptional profiling of dried blood spot samples.^27,28^

### Statistical Analysis

As in previous dried blood spot CTRA studies, we used linear mixed effect models to analyze average expression of a pre-specified set of CTRA indicator gene transcripts as a function of psychosocial risk and resilience factors while controlling for covariates. Analyses focused on a pre-specified set of 53 CTRA indicator genes used in previous research,^4,29^ of which 43 were reliably detectable in this study, including 16 pro-inflammatory gene transcripts (*CXCL8, FOS, FOSB, FOSL2, IL1B, JUN, JUNB, JUND, NFKB1, NFKB2, PTGS1, PTGS2, REL, RELA, RELB, TNF*) and 27 Type I interferon-related gene transcripts (*GBP1, IFI16, IFI27, IFI27L2, IFI35, IFI44, IFI44L, IFI6, IFIH1, IFIT1-IFIT3, IFIT1B, IFIT5, IFITM1-IFITM3, IRF2, IRF7, IRF8, JCHAIN, MX1-MX2, OAS1-OAS3, OASL*), and 10 of which were removed due to minimal expression levels or variation (SD < .5 log2 expression units; *FOSL1, IFI27L1, IFI30, IFITM4P, IFITM5, IFNB1, IGLL1, IGLL3P, ILA1, IL6*). Genespecific z-score signs were reversed for the antiviral gene set to reflect its inverse contribution to the CTRA profile.^4^ Mixed models were estimated by maximum likelihood (SAS PROC MIXED) and specified fixed effects of indicator gene (repeated measure), cognitive status (normal vs mild cognitive impairment), psychosocial risk/resilience factors, a cognitive status x psychosocial factor interaction term (testing for differences in CTRA association as a function of cognitive status), and covariates (age, sex, race, BMI, history of regular smoking, and history of regular heavy alcohol consumption); a random effect of study participant; and a fully saturated (unstructured) variance-covariance matrix to account for residual heteroscedasticity and correlation across participants. In the event of a significant cognitive status x psychosocial factor interaction, additional follow-up “simple slopes” analyses quantified the association of psychosocial factors with CTRA gene expression nested within cognitive status group.

## Results

### Demographics and Cognitive Status

A total of 171 participants provided dried blood spot samples (106 NC, 58 MCI, 1 Dementia, 6 Other/NA) with 148 of those samples (87%) yielding valid RNA data. Participants without diagnoses of NC or MCI were excluded from further analysis (4), and 1 participant completed dried blood spot without questionnaires and was excluded from further analysis, yielding a final analytic sample of 143 participants: 91 with normal cognition and 52 with MCI. The mean age of our group was 72.9 +/− 8.04 years, 16% were Black, and 69% were female, and 18% were treated with a beta-blocker. Participant demographic characteristics are summarized in Table 1. Less than 4% of data were missing for all variables analyzed.

### Cognitive Impairment and the Psychosocial Correlates of CTRA

To determine how cognitive impairment might affect the relationship between psychosocial factors and CTRA gene expression, we compared the relation of CTRA gene expression to psychosocial risk factors (stress, loneliness), two distinct domains of well-being (hedonic and eudaimonic well-being), and three distinct domains of coping (blame and disengagement, distraction and reframing, and social support) for NC and MCI groups while controlling for covariates.

Consistent with previous reports,^11,30,31^ CTRA gene expression was significantly associated with the 2-dimensional representation of well-being (distinct hedonic and eudaimonic dimensions; F(2, 129) = 4.93, *p* = 0.009; Table 2, Model 1), with a significant inverse association with eudaimonic well-being (−0.045 log_2_ RNA per well-being SD ± 0.015 SE, *p* = 0.003). There was no significant association with hedonic well-being (+0.019 ± 0.015, *p* = 0.225). Neither stress nor loneliness showed any significant association with CTRA gene expression in this sample (F(2, 130) = 0.37, *p* = 0.693; individual *p*’s > 0.50; Table 2, Model 2).

**Figure 1.**
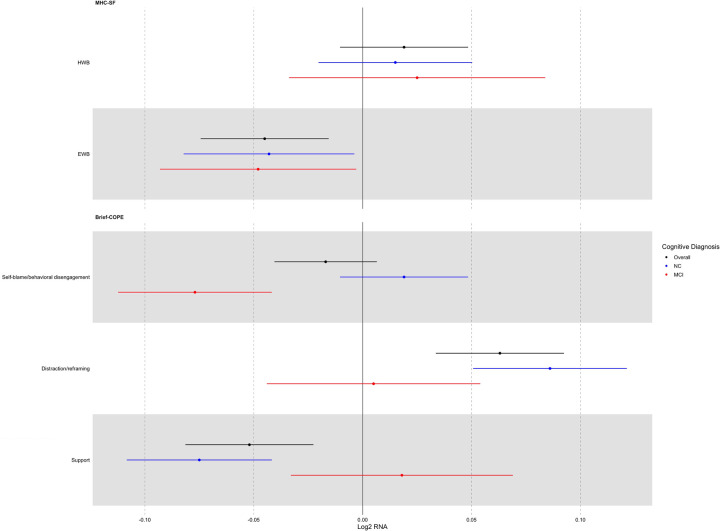
Interactions between cognitive diagnosis and well-being, coping factors. Forest plot demonstrating the strength of association (b ± SE) between the indicated predictor variable and the 53-gene CTRA contrast score for two dimensions of well-being and three coping factors.

CTRA gene expression also varied significantly as a function of the three major dimensions of coping in this sample (F(3, 126) = 7.22, *p* < 0.001; Table 2 Model 3, Figure 1). However, we detected significant interactions between cognitive status and the brief-COPE as it relates to CTRA gene expression (F(3, 123) = 9.07, *p* < 0.001). Among those with normal cognitive function, coping through social support was associated with lower CTRA gene expression (−0.075 ± 0.017, *p* < 0.001) whereas coping by distraction / reframing was associated with higher CTRA gene expression (+0.086 ± 0.018, *p* < 0.001). Among those with MCI, coping by blame or disengagement was associated with a lower CTRA gene expression (−0.077 ± 0.018, *p* < 0.001).

CTRA gene expression was significantly associated in a model containing both psychosocial risk factors (perceived stress, loneliness) and dimensions of well-being (eudaimonic, hedonic); F(2, 126) = 11.72, *p* < 0.001; Table 2, Model 4), with a significant inverse relationship between CTRA gene expression and eudaimonic well-being (−0.076 ± 0.016, *p* < 0.001) and loneliness (−0.050 ± 0.015, *p* < 0.001) with no effect modification by cognitive diagnosis. There are significant correlations between eudaimonic well-being and hedonic well-being (R = 0.60), loneliness (R = −0.59), and perceived stress (R = −0.42).

A model including both eudaimonic well-being and the three coping factors was significantly associated with CTRA gene expression; F(3, 123) = 7.01, *p* < 0.001 (Table 2, Model 5). In this model, there was a significant inverse correlation between CTRA gene expression and eudaimonic well-being (−0.03 ± 0.012, *p* = 0.013) with no effect modification of cognitive diagnosis. However, we again detected significant interactions between cognitive status and the brief-COPE in relation to CTRA gene expression (F(3, 119) = 10.71, *p* < 0.001). Among those with normal cognitive function, coping through social support was associated with a lower CTRA gene expression (−0.076 ± 0.017, *p* < 0.001) whereas coping by distraction / reframing was associated with a higher CTRA gene expression (+0.091 ± 0.017, *p* < 0.001). Among those with MCI, coping with blame or disengagement was associated with a lower CTRA gene expression (−0.080 ± 0.018, *p* < 0.001). There was no significant correlation between eudaimonic well-being and these coping factors.

Several COVID-19-related factors were associated with significant differences in CTRA gene expression, none of which differed by cognitive diagnosis (F(3, 124) = 3.20, p = 0.026). A past diagnosis of COVID-19, either confirmed or suspected, was associated with a lower CTRA gene expression (−0.144 ± 0.058, *p* = 0.014). Of note, only 6 out of 143 (4%) of our participants reported a past COVID diagnosis, and the timing of past infection was not documented. Participants who reported feeling isolated due to COVID-19 had a lower CTRA gene expression (−0.027 ± 0.011, *p* = 0.019), and those who reported higher distress had a higher CTRA gene expression (+0.030 ± 0.018, *p* = 0.019). Degree of worry about COVID-19 was not significantly associated with CTRA gene expression (+0.024 ± 0.015, *p* = 0.105).

## Discussion

Our analysis of genome regulation in the context of the COVID-19 pandemic during the period of general social distancing documented distinctive transcriptional correlates of wellbeing and dimensions of coping. In both MCI and NC, these data are consistent with previous research in identifying an inverse association of CTRA gene expression with eudaimonic well-being. For the NC group, CTRA gene expression was also inversely associated with coping through social support, but directly (unfavorably) associated with coping by distraction and reframing. By contrast, CTRA gene expression was not associated with either of those coping dimensions for individuals with MCI. The patterns of similar and distinct associations for MCI vs NC suggests that broad experiences of psychological and social well-being remain centrally relevant to biobehavioral function in the context of MCI, whereas more specific dimensions of self-management and coping may become less relevant to individual biobehavioral function in the context of MCI as individuals come to depend more on others to help support activities of daily life and cope with challenge, and thus less predominately dependent on their own cognitive processes and coping responses.

High levels of loneliness have been shown to be associated with an upregulation of CTRA gene expression.^32^ However, loneliness did not predict CTRA profile in our cohort. It is possible that the relatively low loneliness scores among our participants was below a threshold at which an effect would be seen. One previous study found that eudaimonic wellbeing had a stronger relationship with CTRA gene expression than did loneliness when both variables are considered simultaneously, suggesting that the two variables’ effects may stem from their common involvement in social well-being.^33^ In a model containing both eudaimonic well-being an loneliness, we found that eudaimonic retained a significant inverse association with CTRA gene expression but a counterintuitive inverse relationship between loneliness and CTRA gene expression appeared after the shared variance between these two variables of interest was accounted for, a finding that will need to be explored in future work. Previous studies of the association of eudaimonic and hedonic well-being with CTRA profiles have demonstrated similar findings to that of our cohort. In a study of 84 healthy adults aged 35 – 64, eudaimonic well-being was associated with downregulated CTRA gene expression, while hedonic well-being was associated with CTRA upregulation.^30^

Wyman and colleagues^34^ recently reported on the association between psychological wellbeing as measured in the NIH Toolbox on Emotion and found that, in a race-stratified analysis, Black and American Indian/Alaskan Native participants reported lower life satisfaction than White participants, but similar scores on positive affect, meaning in life, and purpose in life. Measures of executive functions, but not episodic memory, were higher in those with higher life satisfaction scores. Psychological well-being is a multi-dimensional construct, and includes evaluative well-being related to evaluations made about life, hedonic well-being or pleasures and satisfaction from life, and eudaimonic well-being or a sense of greater purpose in life.^35^ Subjective clinical complaints associated with MCI, such as memory concerns, are predictive of reduced psychological well-being in individuals.^36^ Additionally, neuropsychiatric symptoms in MCI are associated with increased risk of incident dementia, independent of prior functional or cognitive status.^37^ It is possible that subjective clinical complaints and neuropsychiatric symptoms associated with MCI lead to the observed reduction in eudaimonic well-being among MCI participants in this study. Interventions targeting subjective self-reported health and emotional factors related to well-being have the potential to improve eudaimonic well-being and reduce the associated upregulation in CTRA profile.

The default mode network, an intrinsic connectivity network that is central to AD pathogenesis,^38^ has been implicated in the neural correlates of loneliness and a sense of meaning and purpose in life (a component of eudaimonic well-being).^39^ Internetwork connectivity was more dense and less modular between the default mode network, and the frontoparietal, attention, and perceptual networks in lonely individuals. Conversely, a greater sense of meaning in life was associated with an increase in modularity between the default mode and limbic networks. This work suggests that the default mode network is a central hub that is involved in shifting between states through differences in modularity and integration with the frontoparietal and limbic networks. These data suggest that the benefits of eudaimonic well-being translate to older populations with normal cognition as well as those with MCI. Interventions shown to improve eudaimonic well-being and reduce CTRA gene expression, such as mindfulness meditation practices,^31^ might be investigated in future studies with NC and MCI individuals to gauge improvements in eudaimonic well-being and corresponding CTRA profiles.

Strategies used for coping with psychosocial stress in MCI have been assessed in prior work, though this is the first study to evaluate its molecular correlates in gene expression. Coin and colleagues^40^ assessed coping strategies in people living with MCI and dementia. This study utilized the COPE, but used *a priori* categories of coping, including five scales of problem focused coping, five scales of emotion-focused coping, and three scales of coping strategies sometimes considered less healthy (venting of emotions, behavioral disengagement, and mental disengagement). They found that individuals with greater cognitive impairment had poorer coping strategies. This association remained present even after adjusting for prepandemic depression, suggesting that less efficient coping strategies may have exposed those with a greater degree of cognitive impairment to more psychosocial stress related to pandemic-related social distancing. Murukesu and colleagues^41^ assessed coping strategies using the brief-COPE in older adults with cognitive frailty during the period of restricted movement, travel, and assembly in Malaysia which looked at well-being and coping between two groups in a randomized control trial of a multi-domain intervention to address cognitively frailty. They found that older adults with cognitive frailty used religion, acceptance, and positive reframing (i.e., active coping) and self-blame, denial, and substance use (i.e., avoidant coping) were the least common. Our study adds to this literature in defining the molecular correlates of coping in the context of immune cell gene expression. We also did not utilize *a priori* assumptions of coping strategies and rather relied on a datadriven approach to evaluate coping in our cohort. In our sample, we found that only those with normal cognition demonstrated a reduction in CTRA gene expression with the use of social support. At the same time, among those with normal cognition, coping based on distraction and reframing was associated with elevated CTRA gene expression. One possibility that is beyond the scope of our data to answer is that, as individuals develop prodromal cognitive decline (i.e., MCI), self-appraised coping strategies may become less clearly associated with actual coping strategies. A similar pattern is seen in the self-appraisal of cognitive impairment, where those with MCI demonstrate a progressive underappreciation of their own cognitive deficits.^42^ Given that possibility, it becomes all the more remarkable that eudaimonic well-being remains an important correlate of molecular well-being for adults with MCI as well as for adults with normal cognition, and that consummatory sources of hedonic wellbeing remain a risk even in the context of MCI. Again, this pattern could potentially reflect that persons with MCI may be “outsourcing” their coping to their caregivers/support network, and thus their own psychological reactions bear little relationship to their CTRA biology whereas their engagement with others (social well-being) is the primary psychosocial source of biological resilience.

Several issues limit the interpretation of the present results. In this sample, people with cognitive impairment were both older and lonelier than those without cognitive impairment, and this range-restriction could have contributed to the lack of association observed for loneliness and CTRA gene expression among those with MCI. The present results come from data collected during the COVID-19 pandemic and associated social distancing protocols, and it is unclear whether similar results would be obtained in other settings and social conditions. Our MCI sample was smaller than our NC sample, potentially leading to asymmetric power across subgroups. Because our data come from a single regional context, it is unclear whether our findings would hold true across all individuals with MCI, and future work should focus on larger and more broadly representative samples.

Despite these limitations, our work demonstrates several important findings. This is the first study to demonstrate the similar transcriptional correlates of eudaimonic vs. hedonic wellbeing in individuals with MCI compared to NC individuals. It is well-established that individuals with greater eudaimonic well-being (i.e., a sense of purpose in life) demonstrate a reduced CTRA gene expression profile,^30,31,33^ and past work has found a significant reduction in the risk of AD and MCI associated with a greater sense of purpose in life.^13^ The findings here suggest one potential mechanism through which this psychological resiliency factor may function, mediating a lower inflammatory burden and protecting against the “inflammaging” that has been proposed to contribute to the AD neuropathological cascade.

## Figures and Tables

**Table 1:** Demographics

	NC		MCI		
	(n=91)		(n=52)		*P*-value
	*n*	*%*	*n*	*%*	
Black Participants	16	18	7	13	0.935
Women	67	74	32	62	0.134
Alcohol use	60	66	28	54	0.131
Tobacco use	4	4	3	6	0.715
Beta-Blocker use	13	15	12	24	0.201
	*mean*	*(SD)*	*mean*	*(SD)*	
Age, years	71.4	8.4	75.7	7.4	**0.002**
Education, years	16.2	2.3	15.4	2.5	0.088
BMI	27.7	6.3	28.2	5.5	0.658
MoCA score, total	27.3	2.5	23.0	3.4	< **0.001**
Loneliness	31.8	8.8	35.8	10.4	**0.017**
Perceived stress	5.1	3.2	4.9	2.8	0.694
Hedonic well-being	12.5	2.6	12.2	2.4	0.455
Eudaimonic well-being	42.6	7.8	39.5	9.5	**0.042**
Coping - support factor	2.9	1.3	2.6	1	**0.021**
Coping - distraction and reframing factor	3.0	0.7	2.7	0.8	**0.033**
Coping–behavioral disengagement factor	1.3	0.4	1.3	0.5	0.956

NC = normal cognition; MCI = mild cognitive impairment; MoCA = Montreal Cognitive Assessment

**Table 2:** CTRA relationship to well-being, stress, loneliness, and coping factors

	Model 1			Model 2			Model 3			Model 4			Model 5		
Variables	b	SE	P	b	SE	P	b	SE	P	b	SE	P	b	SE	P
Eudaimonic well-being	−0.045	0.015	**0.023**							−0.076	0.016	**< 0.001**	−0.030	0.012	**0.013**
Hedonic well-being	0.019	0.015	0.225							0.014	0.016	0.374			
Perceived Stress				0.001	0.013	0.939				−0.004	0.014	0.784			
Loneliness				−0.011	0.013	0.417				−0.050	0.015	**< 0.001**			
Self-blame/behavioral disengagement coping factor							−0.168	0.012	0.161*				−0.022	0.012	0.067*
Distraction/reframing coping factor							0.063	0.015	**< 0.001** *				0.062	0.015	**< 0.001** *
Support coping factor							−0.052	0.015	**< 0.001** *				−0.045	0.015	**0.003** *

* denotes a significant interaction with cognitive status.

## References

[1] Albert MS, DeKosky ST, Dickson D, The diagnosis of mild cognitive impairment due to Alzheimer’s disease: recommendations from the National Institute on Aging-Alzheimer’s Association workgroups on diagnostic guidelines for Alzheimer’s disease. Alzheimers Dement 2011;7(3):270–9. 21514249 10.1016/j.jalz.2011.03.008 PMC3312027

[2] McKhann GM, Knopman DS, Chertkow H, The diagnosis of dementia due to Alzheimer’s disease: recommendations from the National Institute on Aging-Alzheimer’s Association workgroups on diagnostic guidelines for Alzheimer’s disease. Alzheimers Dement 2011;7(3):263–9. 21514250 10.1016/j.jalz.2011.03.005 PMC3312024

[3] Grodstein F, Wang T, Leurgans SE, Modifiable psychosocial risk factors and delayed onset of dementia in older populations: analysis of two prospective US cohorts. BMJ Open 2022;12(4):e059317. 10.1136/bmjopen-2021-059317 PMC8981290 35379643

[4] Cole SW. The Conserved Transcriptional Response to Adversity. Current Opinion in Behavioral Sciences 2019;28:31–37. 31592179 10.1016/j.cobeha.2019.01.008 PMC6779418

[5] Cole SW. Human social genomics. PLoS Genet 2014;10(8):e1004601. 25166010 10.1371/journal.pgen.1004601 PMC4148225

[6] Walker KA. Inflammation and neurodegeneration: chronicity matters. Aging (Albany NY) 2018;11(1):3–4. 30554190 10.18632/aging.101704 PMC6339781

[7] Schottenfeld D, Beebe-Dimmer J. Chronic inflammation: a common and important factor in the pathogenesis of neoplasia. CA Cancer J Clin 2006;56(2):69–83. 16514135 10.3322/canjclin.56.2.69

[8] Lopez-Candales A, Hernández Burgos PM, Hernandez-Suarez DF, Harris D. Linking Chronic Inflammation with Cardiovascular Disease: From Normal Aging to the Metabolic Syndrome. J Nat Sci 2017;3(4):e341. 28670620 PMC5488800

[9] Reed RG. Stress and Immunological Aging. Curr Opin Behav Sci 2019;28:38–43. 31179376 10.1016/j.cobeha.2019.01.012 PMC6548512

[10] Cole SW, Hawkley LC, Arevalo JM, Social regulation of gene expression in human leukocytes. Genome Biol 2007;8(9):R189. 17854483 10.1186/gb-2007-8-9-r189 PMC2375027

[11] Fredrickson BL, Grewen KM, Algoe SB, Psychological well-being and the human conserved transcriptional response to adversity. PLoS One 2015;10(3):e0121839. 25811656 10.1371/journal.pone.0121839 PMC4374902

[12] Lamers SMA, Westerhof GJ, Bohlmeijer ET, Evaluating the psychometric properties of the mental health Continuum-Short Form (MHC-SF). Journal of Clinical Psychology 2011;67(1):99–110. 20973032 10.1002/jclp.20741

[13] Boyle PA, Buchman AS, Barnes LL, Bennett DA. Effect of a purpose in life on risk of incident Alzheimer disease and mild cognitive impairment in community-dwelling older persons. Arch Gen Psychiatry 2010;67(3):304–310. 20194831 10.1001/archgenpsychiatry.2009.208 PMC2897172

[14] Coffin C, Suerken CK, Bateman JR, Vascular and microstructural markers of cognitive pathology. Alzheimer’s & Dementia: Diagnosis, Assessment & Disease Monitoring 2022;14(1):e12332. 10.1002/dad2.12332 PMC9257520 35814618

[15] Petersen RC, Morris JC. Mild Cognitive Impairment as a Clinical Entity and Treatment Target. Archives of Neurology 2005;62(7):1160–1163. 16009779 10.1001/archneur.62.7.1160

[16] Cohen S, Kamarck T, Mermelstein R. A global measure of perceived stress. J Health Soc Behav 1983;24(4):385–96. 6668417

[17] Russell DW. UCLA Loneliness Scale (Version 3): reliability, validity, and factor structure. J Pers Assess 1996;66(1):20–40. 8576833 10.1207/s15327752jpa6601_2

[18] Carver CS. You want to measure coping but your protocol’s too long: consider the brief COPE. Int J Behav Med 1997;4(1):92–100. 16250744 10.1207/s15327558ijbm0401_6

[19] Waugh CE, Shing EZ, Furr RM. Not all disengagement coping strategies are created equal: positive distraction, but not avoidance, can be an adaptive coping strategy for chronic life stressors. Anxiety Stress Coping 2020;33(5):511–529. 32329359 10.1080/10615806.2020.1755820

[20] Litman J. The COPE Inventory: Dimensionality and relationships with approach- and avoidance-motives and positive and negative traits. Personality and Individual Differences 2006;41:273–284.

[21] Keyes CLM. The mental health continuum: From languishing to flourishing in life. Journal of Health and Social Behavior 2002;43:207–222. 12096700

[22] Keyes CLM, Shmotkin D, Ryff CD. Optimizing well-being: the empirical encounter of two traditions. J Pers Soc Psychol 2002;82(6):1007–1022. 12051575

[23] Ryff CD, Keyes CLM. The structure of psychological well-being revisited. Journal of Personality and Social Psychology 1995;69:719–727. 7473027 10.1037//0022-3514.69.4.719

[24] COVID-19 V1 | National Alzheimer’s Coordinating Center [Internet]. 2020;[cited 2023 Apr 13] Available from: https://naccdata.org/data-collection/forms-documentation/covid-19-v1

[25] Cawthon P, Orwoll E, Ensrud K, Assessing the impact of the covid-19 pandemic and accompanying mitigation efforts on older adults. J Gerontol A Biol Sci Med Sci 2020; 10.1093/gerona/glaa099 PMC7188163 32307522

[26] Allen AM, Lundeen K, Murphy SE, Web-Delivered Multimedia Training Materials for the Self-Collection of Dried Blood Spots: A Formative Project [Internet]. JMIR Form Res 2018;2(2)[cited 2020 Nov 11] Available from: https://www.ncbi.nlm.nih.gov/pmc/articles/PMC6334672/10.2196/11025 PMC6334672 30684406

[27] McDade TW, Ross K M., Fried R L., Genome-Wide Profiling of RNA from Dried Blood Spots: Convergence with Bioinformatic Results Derived from Whole Venous Blood and Peripheral Blood Mononuclear Cells. Biodemography Soc Biol 2016;62(2):182–97. 27337553 10.1080/19485565.2016.1185600 PMC4972449

[28] Ross KM, Cole SW, Carroll JE, Dunkel Schetter C. Elevated pro-inflammatory gene expression in the third trimester of pregnancy in mothers who experienced stressful life events. Brain Behav. Immun. 2019;76:97–103. 30447280 10.1016/j.bbi.2018.11.009 PMC6348125

[29] Nelson-Coffey SK, Fritz MM, Lyubomirsky S, Cole SW. Kindness in the blood: A randomized controlled trial of the gene regulatory impact of prosocial behavior. Psychoneuroendocrinology 2017;81:8–13. 28395185 10.1016/j.psyneuen.2017.03.025

[30] Fredrickson BL, Grewen KM, Coffey KA, A functional genomic perspective on human well-being. Proc Natl Acad Sci U S A 2013;110(33):13684–13689. 23898182 10.1073/pnas.1305419110 PMC3746929

[31] Boyle CC, Cole SW, Dutcher JM, Changes in eudaimonic well-being and the conserved transcriptional response to adversity in younger breast cancer survivors. Psychoneuroendocrinology 2019;103:173–179. 30703712 10.1016/j.psyneuen.2019.01.024

[32] Cole SW, Hawkley LC, Arevalo JMG, Cacioppo JT. Transcript origin analysis identifies antigen-presenting cells as primary targets of socially regulated gene expression in leukocytes. Proceedings of the National Academy of Sciences 2011;108(7):3080–3085. 10.1073/pnas.1014218108 PMC3041107 21300872

[33] Cole SW, Levine ME, Arevalo JM, Loneliness, eudaimonia, and the human conserved transcriptional response to adversity. Psychoneuroendocrinology 2015;62:11–7. 26246388 10.1016/j.psyneuen.2015.07.001 PMC4637182

[34] Wyman MF, Van Hulle CA, Umucu E, Psychological well-being and cognitive aging in Black, Native American, and White Alzheimer’s Disease Research Center participants [Internet]. Frontiers in Human Neuroscience 2022;16[cited 2023 Jan 6] Available from: https://www.frontiersin.org/articles/10.3389/fnhum.2022.924845 10.3389/fnhum.2022.924845 PMC9372578 35967004

[35] Steptoe A, Deaton A, Stone AA. Psychological wellbeing, health and ageing. Lancet 2015;385(9968):640–648. 25468152 10.1016/S0140-6736(13)61489-0 PMC4339610

[36] Gates N, Valenzuela M, Sachdev PS, Singh MAF. Psychological well-being in individuals with mild cognitive impairment. Clin Interv Aging 2014;9:779–792. 24855347 10.2147/CIA.S58866 PMC4020883

[37] Rosenberg PB, Mielke MM, Appleby BS, The association of neuropsychiatric symptoms in MCI with incident dementia and Alzheimer disease. Am J Geriatr Psychiatry 2013;21(7):685–695. 23567400 10.1016/j.jagp.2013.01.006 PMC3428504

[38] Seeley WW, Crawford RK, Zhou J, Neurodegenerative diseases target large-scale human brain networks. Neuron 2009;62(1):42–52. 19376066 10.1016/j.neuron.2009.03.024 PMC2691647

[39] Mwilambwe-Tshilobo L, Ge T, Chong M, Loneliness and meaning in life are reflected in the intrinsic network architecture of the brain. Social Cognitive and Affective Neuroscience 2019;14(4):423–433. 30924854 10.1093/scan/nsz021 PMC6523421

[40] Coin A, Devita M, Trevisan C, Psychological Well-Being of Older Adults With Cognitive Deterioration During Quarantine: Preliminary Results From the GeroCovid Initiative [Internet]. Frontiers in Medicine 2021;8[cited 2022 Nov 11] Available from: https://www.frontiersin.org/articles/10.3389/fmed.2021.715294 10.3389/fmed.2021.715294 PMC8493282 34631737

[41] Murukesu RR, Singh DKA, Shahar S, Subramaniam P. Physical Activity Patterns, Psychosocial Well-Being and Coping Strategies Among Older Persons with Cognitive Frailty of the “WE-RISE” Trial Throughout the COVID-19 Movement Control Order. Clin Interv Aging 2021;16:415–429. 33692620 10.2147/CIA.S290851 PMC7939500

[42] Edmonds EC, Weigand AJ, Thomas KR, Increasing Inaccuracy of Self-Reported Subjective Cognitive Complaints over 24 Months in Empirically-Derived Subtypes of Mild Cognitive Impairment. J Int Neuropsychol Soc 2018;24(8):842–853. 30278855 10.1017/S1355617718000486 PMC6173206

